# Immunoglobulin G Subclass-Specific Glycosylation Changes in Primary Epithelial Ovarian Cancer

**DOI:** 10.3389/fimmu.2020.00654

**Published:** 2020-05-15

**Authors:** Marta Wieczorek, Elena Ioana Braicu, Leticia Oliveira-Ferrer, Jahid Sehouli, Véronique Blanchard

**Affiliations:** ^1^Institute of Laboratory Medicine, Clinical Chemistry and Pathobiochemistry, Charité – Universitätsmedizin Berlin, Corporate Member of Freie Universität Berlin, Humboldt-Universität zu Berlin, and Berlin Institute of Health, Berlin, Germany; ^2^Department of Biology, Chemistry and Pharmacy, Freie Universität Berlin, Berlin, Germany; ^3^Department of Gynecology, Charité – Universitätsmedizin Berlin, Corporate Member of Freie Universität Berlin, Humboldt-Universität zu Berlin, Berlin Institute of Health, NOGGO Group, Berlin, Germany; ^4^Department of Gynecology, University Medical Center Hamburg-Eppendorf, Hamburg, Germany

**Keywords:** IgG subclasses, N-glycopeptides, glycosylation, ovarian cancer, MALDI-TOF-MS

## Abstract

Epithelial ovarian cancer (EOC) was previously shown to be associated with glycosylation changes of total serum and total IgG proteins. However, as a majority of previous studies analyzed released glycan profiles, still little is known about IgG subclass-specific alterations in ovarian cancer. Hence, in this study, we investigated EOC-related glycosylation changes of the three most abundant IgG subclasses, namely, IgG_1_, IgG_2_ and IgG_3_ isolated from sera of 87 EOC patients and 74 age-matched healthy controls. In order to separate IgG_2_ and IgG_3_, we performed a two-step affinity purification employing Protein A and Protein G Sepharose. After tryptic digestion, IgG glycopeptides were enriched and measured by MALDI-TOF-MS. Finally, EOC-related glycosylation changes were monitored at the level of total agalactosylation, monogalactosylation, digalactosylation, sialylation, bisection and fucosylation, which were calculated separately for each IgG subclass. Interestingly, aside from an EOC-related increase in agalactosylation/decrease in monogalactosylation and digalactosylation observed in all IgG subclasses, some subclass-specific trends were detected. Glycosylation of IgG_1_ was found to be most strongly affected in EOC, as it exhibited the highest number of significant differences between healthy controls and EOC patients. Specifically, IgG_1_ was the only subclass that showed a significant decrease in sialylation and a significant increase in fucosylation in EOC patients. Interestingly, IgG_2_ and IgG_3_ that were often investigated collectively in previous studies, were found to have distinct glycosylation patterns. IgG_3_ displayed stronger EOC-related increase in agalactosylation/decrease in digalactosylation and was characterized by notably higher sialylation, which consequently decreased in EOC patients. In conclusion, our study indicates that IgG subclasses exhibit subtly distinct glycosylation patterns of EOC-related alterations and that IgG_1_ and IgG_3_ agalactosylation show the strongest association with CA125, the routine diagnostic marker. Additionally, our results show that simultaneous analyses of IgG_2_ and IgG_3_ might lead to wrong conclusions as these two subclasses exhibit noticeably different glycosylation phenotypes.

## Introduction

Glycosylation of proteins is one of the most common co-translational and post-translational modifications that consists of the covalent attachment of carbohydrate moieties to polypeptide chains. Apart from increasing protein diversity, glycans are known to play a broad range of roles; that is, they govern the folding of nascent polypeptides in the ER, provide protein physicochemical stability and modify their functions. Moreover, they are involved in various biological processes including cell signaling, extracellular interactions and immune responses (1, 2). Consequently, altered protein glycosylation was shown to be associated with numerous inborn and acquired pathological conditions (3, 4).

Among others, glycosylation changes were reported in ovarian cancer (OC), which remains the most lethal gynecological malignancy in women despite decades of research and better understanding of its etiology (5). Its particularly high mortality results from the lack of early diagnostic markers, which leads to late primary diagnosis and 5-year survival rates as low as 29% in advanced-stage patients (6). In the absence of biomarkers able to reliably detect OC at an early, still asymptomatic stage, altered glycosylation of proteins attracted attention as a potential source of complementary screening markers. Hence, in the last decades, OC-related changes in glycosylation of total serum and of individual proteins were broadly investigated by us (7–9) and by other groups (10–13). Since carcinoma development is accompanied by inflammation, we previously analyzed the N-glycosylation profile of acute-phase proteins isolated from serum samples of epithelial ovarian cancer (EOC) patients (14). Obtained glycosylation profiles revealed not only significant quantitative but also qualitative differences between healthy controls and EOC patients. For instance, triantennary N-glycans containing a (1–6) branch and a Lewis^X^ epitope or tetraantennary N-glycans containing three Lewis^X^ epitopes were detected only in EOC patients, which demonstrates their diagnostic potential (14).

In the present study, we aimed at further investigating EOC-related glycosylation changes, turning our focus from liver-originating acute-phase proteins to immunoglobulin G (IgG), secreted by activated plasma B cells. Being present at a concentration of 7–18 mg/ml, IgG represents the most abundant glycoprotein in human serum (15). Each IgG molecule consists of two heavy and two light chains that are covalently linked through disulfide bridges to form a tetrameric Y-shaped structure. Functionally, IgG can be divided into two distinctive parts: fragment antigen binding (Fab), responsible for recognizing and binding to specific antigens, and fragment crystallizable (Fc), which exerts effector functions by interacting with complement, Fc-gamma receptors (FcγRs) and neonatal Fc receptor (FcRn) (16). Within the Fc part, each IgG heavy chain carries a single, highly conserved glycosylation site at asparagine 297 that is typically occupied by biantennary N-glycans of complex type (16, 17). A vast majority of Fc N-glycans is core-fucosylated and bears 0–2 galactose residues. Hence, the three most prominent IgG-Fc glycoforms are G0F (no galactose and one core-fucose), G1F (one galactose and one core-fucose) and G2F (two galactoses and one core-fucose). A small proportion of IgG N-glycans can be further extended by the addition of bisecting *N*-acetylglucosamine (GlcNAc, N) and/or sialic acids (S) (17). Multiple studies showed that IgG-Fc N-glycans are necessary for the maintenance of IgG structural stability, quaternary conformation and induction of its effector functions (18–20).

The presence of aberrantly glycosylated IgG antibodies in the circulation of OC patients was described already in 2001 (21). Later, Saldova et al. (10) defined the nature of these alterations, showing that the serum of OC patients contains a higher fraction of IgG carrying agalactosylated N-glycans and, conversely, lower proportions of those carrying monogalactosylated, digalactosylated and sialylated glycan structures. Their observations, based on the analysis of only three OC serum samples, were further validated in bigger cohort studies by Alley et al. (12) and Qian et al. (13), who not only confirmed that OC was associated with a decreased galactosylation and an increased agalactosylation of IgG but also indicated that observed changes, when combined into a ratio, were superior to routinely used CA125 for discrimination between OC and benign gynecological conditions (13). Based on their results, authors proposed that this ratio could act as an adjunct diagnostic marker, which in combination with CA125 could reduce the number of false-positive outcomes. More recently, Ruhaak et al. examined glycosylation profiles of three major immunoglobulins that are IgG, IgA and IgM, in OC patients, showing that alterations of IgG glycosylation are the most capable of discriminating between OC and healthy individuals (22).

Interestingly, even though human IgG occurs as four subclasses, namely, IgG_1_, IgG_2_, IgG_3_ and IgG_4_, a vast majority of the above-mentioned studies monitored glycosylation changes only at the level of total IgG, analyzing enzymatically released IgG N-glycans. Even though Ruhaak et al. (22) performed the analysis at the level of tryptic glycopeptides, their study concentrated on finding the best-performing OC classifier rather than revealing IgG subclass-specific glycosylation alterations. Uncovering subclass-specific IgG glycosylation profiles is, however, important since, despite 90% identity of their amino acid sequences, each subclass is unique and varies with regard to antigen binding, triggering effector functions, half-life and placental transport. Particular attention should be given to IgG_3_, because its superior affinity to activating FcγRs (i.e., FcγRI, FcγRIIa, FcγRIIIa, and FcγRIIIb) and complement component 1q (C1q) makes it the most potent pro-inflammatory IgG subclass (23). Despite its unique character, analysis of IgG_3_ Fc-glycosylation is rarely performed. This might not only be caused by its lower concentration in blood but also by the fact that tryptic IgG_3_ glycopeptide containing asparagine 297 shares the same peptide backbone as IgG_2_ or IgG_4_, depending on the IgG_3_ allotype (24).

Therefore, the aim of our study was to investigate the subclass-specific distribution of IgG glycosylation changes in patients suffering from primary EOC. By performing two-step affinity purification, glycosylation profiles of the three most abundant IgG subclasses, namely, IgG_1_, IgG_2_ and IgG_3_, could be determined individually.

## Materials and Methods

### Sample Collection

Ethical approval was obtained from the Charité Institutional Ethics Committee (EA4/073/06) and from the Ethics Committee of the Medical Association of Hamburg (#OB/V/03). Written informed consent was obtained from all study participants. Eighty-seven primary EOC patients and 74 age- and sex-matched healthy controls were enrolled in this study (Table 1). Blood withdrawal was performed using clot activator serum tubes (BD Vacutainer, NJ, USA). At first, blood was allowed to clot for 30 min to 2 h at room temperature and then serum was separated by centrifugation at 1,200 g for 15 min. The obtained serum was aliquoted and stored at −80°C until the time of further analysis.

**TABLE 1:** Demographics of the cohorts used in this study.

	**Healthy**	**EOC**
No of patients	74	87
Age (mean ± SD)	59.8 ± 11.2	59.5 ± 10.3
CA125 (mean ± SD)	37.2 ± 106.8	929.4 ± 1,482.0

*Age is shown in years and CA125 values are shown as kU/L.*

### IgG Purification

IgG was isolated from 5 μl of human serum by sequential two-step affinity purification using immobilized Protein A and immobilized Protein G, as described elsewhere (24), with some modifications. Briefly, 30 μl of Protein A Sepharose CL-4B slurry (GE Healthcare, Eindhoven, The Netherlands) was transferred to a 1.5-ml tube containing 400 μl of phosphate-buffered saline (1 × PBS). After the beads settled, the supernatant was discarded and replaced with 400 μl of 1 × PBS, which was repeated one more time. Following washing, the volume was brought up to 400 μl with 1 × PBS and 5 μl of blood serum were added. The sample mixture was incubated under agitation at 650 rpm for 1 h at room temperature. The entire mixture was applied into a shortened 200-μl filter tip. The flow-through was collected and Protein A Sepharose beads were washed with 5 × 200 μl of 1 × PBS and 3 × 200 μl of Milli-Q water. Captured IgG_1_, IgG_2_ and IgG_4_ were eluted with 3 × 100 μl of 100 mM formic acid (Honeywell Fluka, Steinheim, Germany) and evaporated to dryness in a vacuum centrifuge. Protein A Sepharose beads were regenerated by washing with 2 × 100 μl of 100 mM formic acid, 2 × 200 μl of Milli-Q water and 2 × 200 μl of 1 × PBS. The remaining supernatant was then reapplied on the regenerated Protein A Sepharose column and the flow-through was collected. Protein G Sepharose (30 μl of slurry, GE Healthcare, Eindhoven, The Netherlands) was conditioned as described above for Protein A Sepharose and resuspended in the Protein A flow-through. The following incubation, washing and elution steps were performed the same way as described above for Protein A Sepharose. Collected eluates were dried in vacuum centrifuge and stored at −20°C until further analysis.

### SDS-PAGE and Western Blot

Protein A-bound IgG_1_, IgG_2_ and IgG_4_ fraction as well as Protein G-bound IgG_3_ fraction were analyzed by sodium dodecyl sulfate polyacrylamide gel electrophoresis (SDS-PAGE) followed by Coomassie Brilliant Blue staining and western blot analysis using a polyvinylidene fluoride (PVDF) membrane, all using standard techniques. SDS-PAGE was performed using 10% acrylamide gel under reducing conditions. The presence of IgG_3_ in Protein A- and Protein G Sepharose-bound fractions was assessed by western blot using recombinant anti-human IgG_3_ antibody (1:1,000, Abcam, Cambridge, England).

### Tryptic Digestion

Dried IgGs were dissolved in 50 μl of 50 mM ammonium bicarbonate (Merck, Darmstadt, Germany) and shaken for 5 min. Lyophilized sequencing-grade modified trypsin (20-μg vial; Promega, Madison, WI) was dissolved in 100 μl of ice-cold resuspension buffer provided by the manufacturer to obtain a final concentration of 0.2 μg/μl. Five microliters of the solution, corresponding to 1 μg of trypsin, were added to each IgG sample, followed by overnight incubation at 37°C. On the following day, digested IgGs were dried out in a vacuum centrifuge and stored at −20°C until cotton HILIC purification.

### Cotton HILIC Purification

IgG glycopeptides were enriched using self-made micro-spin cotton HILIC columns as described elsewhere (25), with minor modifications. Briefly, 10-μl filter tips (Greiner Bio-One, Kremsmünster, Austria) were filled with fibers of cotton wool pads and conditioned by washing with 3 × 50 μl of Milli-Q water and 3 × 50 μl of 80% ACN. Afterward, dried IgG samples were resuspended in 50 μl of 80% ACN and loaded on the self-made microcolumns. They were washed with 3 × 50 μl of 80% ACN containing 0.1% TFA and then with 3 × 50 μl of 80% ACN. The retained IgG glycopeptides were eluted with 6 × 50 μl Milli-Q water, dried out in a vacuum centrifuge and stored at −20°C until MALDI-TOF-MS measurements.

### MALDI-TOF-MS Measurements and Data Processing

Dried IgG glycopeptide samples were dissolved in Milli-Q water, and 1 μl was spotted on the stainless-steel MALDI target plate (Bruker Daltonics, Bremen, Germany). Once dried, the sample was overlaid with 1 μl of 2.5 mg/ml 4-chloro-α-cyanocinnamic acid (ClCCA, Sigma-Aldrich, Germany) in 70% ACN and allowed to air-dry at room temperature. All measurements were performed on an Ultraflex III mass spectrometer (Bruker Daltonics, Bremen, Germany) equipped with a Smart Beam laser (laser frequency 100 Hz). Prior to measurement, the mass spectrometer was calibrated with Peptide Calibration Standard II (Bruker Daltonics, Bremen, Germany). Spectra were recorded in reflectron negative ionization mode within the *m*/*z* window from 1,000 to 5,000. For each mass spectrum, 5,000 laser shots were accumulated using a partial “random-walk” laser movement mode. Raw mass spectra were exported as ASCII text files, and the subsequent processing that included recalibration, baseline subtraction and peak extraction was performed with the MassyTools software (26). Recalibration was performed using the list of six IgG_1_ glycopeptides (G0F, G1F, G0FN, G2F, G1FN and G2FS1) for mass spectra of Protein A Sepharose-bound IgG or the list of six IgG_3_ glycopeptides (G0F, G1F, G0FN, G2F, G1FN and G2FS1) for mass spectra of Protein G Sepharose-bound IgG. The absolute intensities of the detected glycopeptides were normalized to the total area for IgG_1_, IgG_2_ and IgG_3_. Then, by summing up relative intensities of respective glycopeptide structures, six (or five in case of IgG_2_) derived glycosylation traits, namely, agalactosylation, monogalactosylation, digalactosylation, sialylation, bisecting GlcNAc and fucosylation, were calculated separately for each IgG subclass, according to the formulas listed below:

Agalactosylation (Agal) = G0F + G0FN + G0 + G0N + mono G0F;
Monogalactosylation (Monogal) = G1F + G1FN + G1FS1 + G1 + G1N + G1S1 + mono G1F;
Digalactosylation (Digal) = G2F + G2FN + G2FS1 + G2 + G2N + G2S1;
Sialylation (Sial) = G1FS1 + G2FS1 + G1S1 + G2S1;
Bisecting GlcNAc (Bisec) = G0FN + G1FN + G2FN + G0N + G1N + G2N;
Fucosylation (Fuc) = G0F + G1F + G2F + G0FN + G1FN + G2FN + G1FS1 + G2FS1 + mono G0F + mono G1F.
Fucosylation of IgG_2_ was not evaluated in this study, as most of its afucosylated glycopeptides could not be determined due to the *m/z* overlap with fucosylated glycopeptides of IgG_4_. Schematic representation of all detected structures can be found in Table 2.

**TABLE 2:** Tryptic glycopeptides of human IgG_1_, IgG_2_ and IgG_3_ detected in this study.

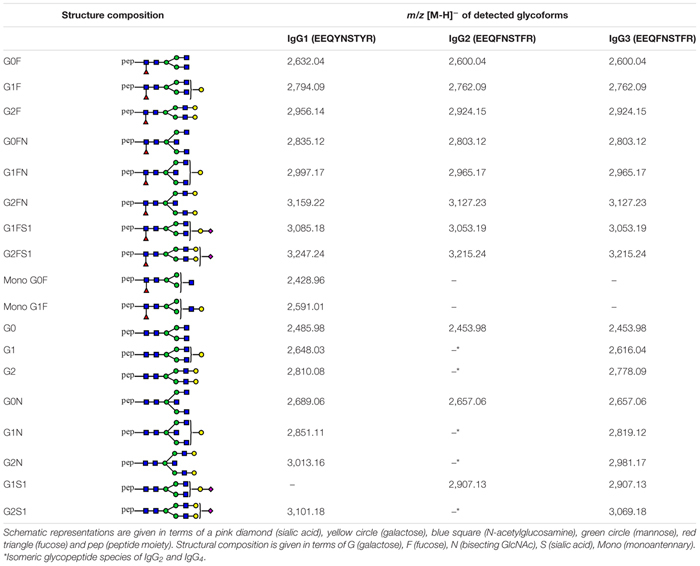	

To determine inter-day reproducibility of an applied workflow, the entire analytical procedure, including two-step IgG purification, tryptic digestion, cotton HILIC enrichment, and MALDI-TOF-MS measurement, was repeated using the same serum sample on three consecutive days. Mean, SD and CV values were calculated for six of the most abundant glycopeptide structures of IgG_1_, IgG_2_ and IgG_3_. Results of reproducibility testing are presented in Supplementary Figure S2.

### Statistical Analysis

Statistical analyses were performed with SPSS version 25.0 (SPSS Inc., Chicago, IL). Means and SD values of IgG_1_, IgG_2_ and IgG_3_ glycosylation traits in healthy controls and EOC patients were calculated and are shown in Supplementary Table S1 and, in a form of bar graphs, in Figure 2. In order to assess associations between IgG glycosylation traits and EOC and to test whether detected EOC-related differences were statistically significant, a regression model was generated for each glycosylation trait. To minimize the effect of age on IgG glycosylation (27, 28), all regression models were corrected by including age as a covariate, which resulted in the following formulas: “glycosylation trait Y ∼ β_1_^∗^ patient’s status (0 = healthy control, 1 = EOC patient) + β_2_^∗^ age + error”. Descriptive statistics of all regression models, that is, regression beta coefficients (β), 95% confidence intervals (95% CI) and *p*-values, are listed in Table 3. In the following parts of this work, β_1_ and *p*_1_ values correspond to EOC-related association, whereas β_2_ and *p*_2_ indicate age-related association. After Bonferroni correction for glycosylation traits, *p*-values smaller than or equal to 0.008 (for IgG_1_ and IgG_3_; 0.05/6 glycosylation traits) and 0.01 (for IgG_2_; 0.05/5 glycosylation traits) were considered as statistically significant. Additionally, for each IgG subclass, the relation between agalactosylation and CA125 diagnostic marker (log-transformed values) was assessed by fitting a linear regression model according to the formula “IgG agalactosylation ∼ β ^∗^ CA125 + error”. Linear graphs with coefficient of determination (*R*^2^), β coefficients and *p*-values are presented in Figure 3. For each regression model, the distribution of residuals was inspected and found to be normal.

**TABLE 3:** Associations between IgG glycosylation traits, EOC and age.

**Glycosylation trait**		**IgG1**			**IgG2**			**IgG3**		
		**β**	**95% CI**	***p***	**β**	**95% CI**	***p***	**β**	**95% CI**	***p***
Agal	(1) EOC	11.285	7.831, 14.738	**1.3E**−**9**	6.056	2.737, 9.374	**4.2E**−**4**	9.120	5.242, 12.998	**7.6E**−**6**
	(2) Age	0.418	0.256, 0.580	**9.9E**−**7**	0.524	0.369, 0.680	**4.4E**−**10**	0.609	0.428, 0.789	**5.7E**−**10**
Monogal	(1) EOC	−5.690	−7.630, −3.750	**3.6E**−**8**	−3.591	−5.666, −1.516	**8.0E**−**4**	−3.516	−5.369, −1.663	**2.6E**−**4**
	(2) Age	−0.118	−0.209, −0.027	0.011	−0.260	−0.357, −0.163	**4.2E**−**7**	−0.162	−0.249, −0.076	**3.0E**−**4**
Digal	(1) EOC	−5.596	−7.466, −3.726	**2.0E**−**8**	−2.465	−3.927, −1.003	**1.1E**−**3**	−5.604	−8.216, −2.992	**4.0E**−**5**
	(2) Age	−0.299	−0.387, −0.212	**2.8E**−**10**	−0.264	−0.333, −0.196	**2.3E**−**12**	−0.446	−0.568, −0.325	**2.5E**−**11**
Sial	(1) EOC	−0.576	−0.949, −0.202	**0.003**	−0.052	−0.644, 0.539	0.861	−2.360	−4.229, −0.492	0.014
	(2) Age	−0.039	−0.057, −0.022	**1.9E**−**5**	−0.072	−0.100, −0.044	**8.3E**−**7**	−0.275	−0.362, −0.188	**4.6E**−**9**
Bisec	(1) EOC	−0.852	−2.141, 0.437	0.193	−0.774	−1.764, 0.215	0.124	−0.564	−1.517, 0.388	0.244
	(2) Age	0.052	−0.008, 0.112	0.091	0.038	−0.009, 0.084	0.110	0.084	0.040, 0.128	**2.7E**−**4**
Fuc	(1) EOC	1.025	0.403, 1.648	**0.001**	–	–	–	0.459	−1.081, 1.998	0.557
	(2) Age	0.005	−0.024, 0.034	0.721	–	–	–	0.096	0.024, 0.168	0.009

*For all glycosylation traits, regression beta coefficients (β) are reported together with their 95% confidence interval (95% CI) and respective *p*-values (*p*). Bonferroni corrected *p*-values smaller than or equal to 0.008 (for IgG_1_ and IgG_3_; 0.05/6 glycosylation traits) and 0.01 (for IgG_2_; 0.05/5 glycosylation traits) were considered as statistically significant and are highlighted in bold. Representations of glycosylation traits are given in terms of Agal (agalactosylation), Monogal (monogalactosylation), Digal (digalactosylation), Sial (sialylation), Bisec (bisecting GlcNAc) and Fuc (core-fucosylation).*

## Results

In the present study, we analyzed the glycosylation profile of the three most abundant IgG subclasses, namely, IgG_1_, IgG_2_ and IgG_3_, in serous EOC patients and age-matched healthy controls by means of MALDI-TOF-MS. Glycosylation analysis was performed at the level of tryptic glycopeptides in order to obtain subclass-specific information. For this purpose, IgG was isolated from serum using two-step affinity purification employing, first, Protein A Sepharose that captures IgG_1_, IgG_2_ and IgG_4_, then Protein G Sepharose in order to bind the IgG_3_ contained in the Protein A Sepharose flow-through. Since the amino acid sequence of IgG_2_ and IgG_3_ glycopeptides are quite often identical (24, 29), the complete depletion of IgG_2_ in the first purification step is essential for the subsequent analysis of IgG_3_-specific glycosylation. As depicted in Supplementary Figure S1A, residual IgG_1_, IgG_2_ and IgG_4_ were removed from the flow-through fraction by its repeated loading on the regenerated Protein A column. The applied procedure allowed the complete binding of IgG_1_, IgG_2_ and IgG_4_, leaving only IgG_3_, which is of higher mass, in the flow-through fraction (Supplementary Figure S1B). In addition, the contamination of the Protein A Sepharose-bound fraction with the IgG_3_ subclass was found to be only minimal and hence negligible as judged by western blot analysis (Supplementary Figure S1C).

**FIGURE 1:**
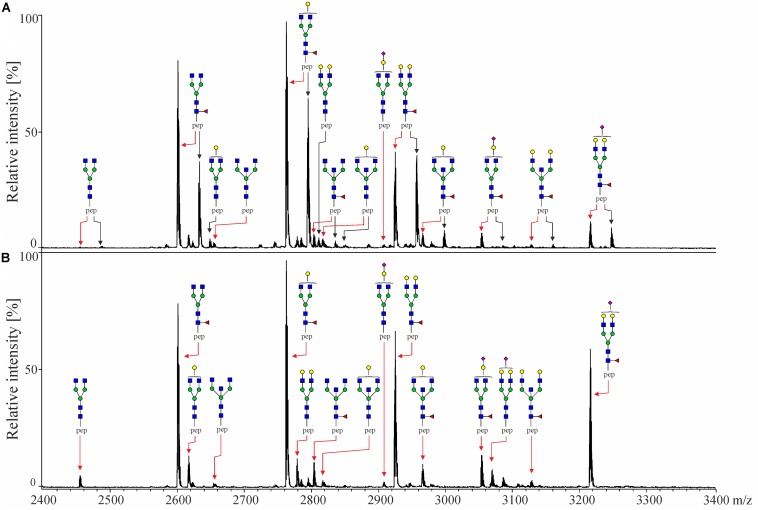
MALDI-TOF mass spectra of tryptic IgG-Fc glycopeptides of **(A)** Protein A Sepharose-bound fraction containing IgG_1_, IgG_2_ and IgG_4_ and **(B)** Protein G Sepharose-bound fraction containing IgG_3_, both obtained from the same healthy individual. Black arrows indicate glycopeptides of IgG_1_, while red arrows indicate IgG_2_ [in panel **(A)**] and IgG_3_ [in panel **(B)**]. IgG_4_ glycopeptides are not indicated as they were not analyzed in this study. Spectra were recorded in the negative-ionization mode. Schematic representations are given in terms of a pink diamond (sialic acid), yellow circle (galactose), blue square (*N*-acetylglucosamine), green circle (mannose), red triangle (fucose) and pep (peptide moiety).

Captured IgG fractions were digested with trypsin, which generates the following peptide sequences: EEQYNSTYR in IgG_1_, EEQFNSTFR in IgG_2_/IgG_3_ and EEQFNSTYR in IgG_4_, where asparagine (N) is occupied by covalently linked N-glycans. Prior to MALDI-TOF-MS measurements, obtained glycopeptides were enriched using self-made cotton HILIC columns (25). Eventually, measurements were performed in negative-ionization mode to enable the simultaneous detection of neutral and underivatized negatively charged (sialylated) IgG glycopeptides. The exemplary MALDI-TOF mass spectra of Protein A- and Protein G-bound fractions are presented in Figure 1, whereas all detected structures included in the IgG glycopeptide analysis are listed in Table 2. In total, we were able to detect 17 peaks corresponding to IgG_1_ glycopeptides, 11 peaks of IgG_2_ glycopeptides and 16 peaks of IgG_3_ glycopeptides. IgG_4_ glycopeptides were not investigated due to very low signals and *m*/*z* overlap with some afucosylated structures of the more abundant IgG_2_ glycopeptides (24). In case of IgG_1_ and IgG_3_, glycopeptides carrying both core-fucosylated and afucosylated N-glycans were analyzed. In turn, due to the above-mentioned *m*/*z* overlap with fucosylated IgG_4_ structures (24), a majority of IgG_2_ glycopeptides carrying afucosylated N-glycans could not be determined and hence were not taken into account during data analysis. Nevertheless, considering that core-fucosylated structures were shown to constitute on average 97% of all IgG_2_ glycoforms (30), exclusion of afucosylated structures has only a minor impact on the entire analysis. Almost all detected glycopeptides carried biantennary complex-type N-glycans bearing zero to two galactoses. Minor amounts of glycopeptides were additionally occupied by bisecting GlcNAc and/or sialic acid.

Since IgG glycosylation is known to vary with age (27, 28), the significance of EOC-related changes was determined with the help of regression models, considering age as a covariate. As visible in Table 3, most of the investigated glycosylation traits indeed showed a significant association with age. Consistent with previous studies (31, 32), in all IgG subclasses, agalactosylation was observed to positively correlate with age, whereas monogalactosylation, digalactosylation and sialylation showed a negative correlation, as judged by their respective β coefficient values. Interestingly, glycosylation of IgG_1_ was found to be the least affected by age, as only three glycosylation traits (agalactosylation, digalactosylation and sialylation) showed a significant association. On the contrary, in the case of IgG_3_, a significant association with age was observed for nearly all glycosylation traits, with fucosylation being the only trait, whose *p*-value (*p*_2_ = 0.009) exceeded the Bonferroni-corrected threshold of α = 0.008 (0.05/6 glycosylation traits), deeming the association insignificant.

### IgG_1_ Profiling

**FIGURE 2:**
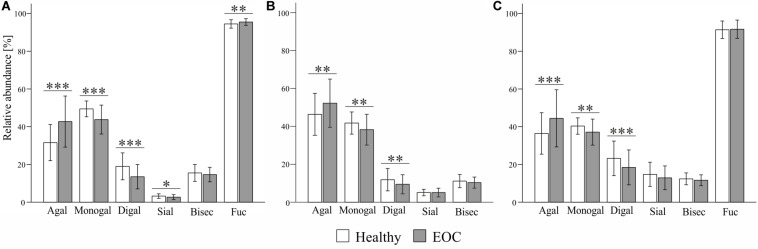
Comparison of **(A)** IgG_1_-, **(B)** IgG_2_- and **(C)** IgG_3_-specific glycosylation profiles between healthy controls and EOC patients. Relative abundance of given glycosylation traits was obtained by summing up relative intensities of the corresponding tryptic glycopeptides detected by MALDI-TOF-MS. Bars represent mean relative abundance and error bars represent standard deviation. *p*-values were obtained from regression analysis, adjusting for age and patients’ status (0 = healthy control, 1 = EOC). After Bonferroni correction for glycosylation traits, statistically significant differences are indicated as ^∗^*p* ≤ 0.008, ^∗∗^*p* ≤ 0.001 and ^∗∗∗^*p* ≤ 0.0001 in case of IgG_1_ and IgG_3_, and ^∗^*p* ≤ 0.01, ^∗∗^*p* ≤ 0.002 and ^∗∗∗^*p* ≤ 0.0002 in case of IgG_2_.

Among all three subclasses, the glycosylation profile of IgG_1_ was found to be the most markedly altered in EOC patients. As visible in Figure 2 and Table 3, IgG_1_ exhibited the most pronounced increase in agalactosylation (β_1_ = 11.285, *p*_1_ = 1.3E−9) and the most pronounced decrease in monogalactosylation and digalactosylation in EOC patients (β_1_ = −5.690, *p*_1_ = 3.6E−8 and β_1_ = −5.596, *p*_1_ = 2.0E−8, respectively). Interestingly, even though IgG_1_ was found to be characterized by generally low sialylation, it was the only subclass in which sialylation was significantly decreased (*p*_1_ = 0.003) in EOC patients. Additionally, IgG_1_ was also found to contain the highest proportion of glycopeptides carrying bisecting GlcNAc (Supplementary Table S1); however, no significant differences were observed between healthy controls and EOC patients. A vast majority (94–96%) of all detected IgG_1_ glycopeptides carried core-fucosylated N-glycans. Remarkably, even though the difference in IgG_1_ core-fucosylation between healthy controls and EOC patients was minor (94.5 ± 2.2 in healthy controls versus 95.5 ± 1.8 in EOC patients), the EOC-related increase was found to be statistically significant (*p*_1_ = 0.001).

### IgG_2_ Profiling

EOC-related glycosylation alterations of IgG_2_ partially resembled those of IgG_1_; however, they were less pronounced (Figure 2 and Table 3). IgG_2_ agalactosylation was significantly increased (β_1_ = 6.056, *p*_1_ = 4.2E−4), whereas monogalactosylation and digalactosylation were significantly decreased in EOC patients (β_1_ = −3.591, *p*_1_ = 8.0E−4 and β_1_ = −2.465, *p*_1_ = 1.1E−3, respectively). Interestingly, IgG_2_ was found to be characterized by particularly high agalactosylation, which was observed in both investigated groups of individuals. For instance, while in IgG_1_ agalactosylation represented 31.6% and 42.7% in healthy controls and EOC patients, respectively, in IgG_2_, these were 46.8% and 52.3%, respectively. Hence, on average, IgG_2_ agalactosylation was about 12.4% higher in each investigated group. This was further reflected by very low IgG_2_ digalactosylation, which constituted only 11.9% in healthy controls and 9.5% in EOC patients (Supplementary Table S1). IgG_2_ sialylation was slightly higher than in IgG_1_; however, no significant differences were detected between healthy controls and EOC patients. Likewise, the abundance of bisecting GlcNAc in IgG_2_ was not found to differ between both investigated groups. As mentioned in previous sections, IgG_2_ fucosylation was not evaluated in this study due to the *m*/*z* overlap with fucosylated IgG_4_ glycopeptides.

### IgG_3_ Profiling

Analysis of IgG_3_ glycosylation is known to be hindered by its high genetic polymorphism (24, 29). In the Caucasian population, tryptic digestion of IgG_3_ usually results in the peptide sequence EEQFNSTFR that is identical to that of IgG_2_ (29). However, due to genetic polymorphism, in some individuals, tyrosine replaces phenylalanine at position 296. In this study, 16 samples (i.e., seven healthy controls and nine EOC patients) were excluded from the analysis of the IgG_3_ glycosylation due to the presence of different peptide sequences and their resulting glycopeptide moieties, leading to normalization issues. Nonetheless, this did not lead to an age mismatch of investigated groups as the mean age ± SD values remained at 61.6 ± 11.2 years in the healthy group and 59.8 ± 10.3 years in the EOC group, with the difference being statistically insignificant (data not shown). As visible in Figure 2 and Table 3, EOC-related glycosylation changes in IgG_3_ resembled those observed in other subclasses; however, they were more pronounced than in IgG_2_ and less pronounced than in IgG_1_. Agalactosylation of IgG_3_ increased in EOC patients (β_1_ = 9.120, *p*_1_ = 7.6E−6), whereas monogalactosylation and digalactosylation decreased (β_1_ = −3.516, *p*_1_ = 2.6E−4 and β_1_ = −5.604, *p*_1_ = 4.0E−5, respectively). Interestingly, IgG_3_ was found to be characterized by markedly higher sialylation than in IgG_1_ and IgG_2_. However, even though its decrease in EOC patients was clearly visible, after Bonferroni correction, the difference between both groups (*p*_1_ = 0.014) was deemed statistically insignificant. Both bisecting GlcNAc and fucosylation of IgG_3_ showed no significant differences between healthy controls and EOC patients.

### Association of IgG Agalactosylation With CA125 Diagnostic Marker

**FIGURE 3:**
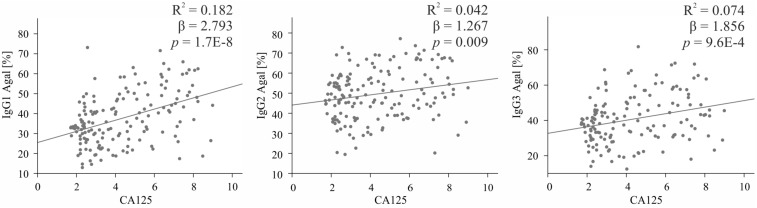
Association between IgG subclass-specific agalactosylation (*Y*-axis) and log (CA125), the routine diagnostic marker (*X*-axis). Descriptive statistics are shown for the total cohort and are given in terms of *R*^2^ (coefficient of determination), β (regression β coefficient) and *p* (*p*-values). IgG_1_ is shown on the **left**, IgG_2_ in the **middle** and IgG_3_ on the **right**.

Among all glycosylation traits, IgG agalactosylation displayed the most profound EOC-related alteration. Thus, its association with the most widely used biomarker for OC, namely, CA125, was assessed in the entire cohort using linear regression models. As visible in Figure 3, in each IgG subclass, agalactosylation showed a significant positive association with CA125. As judged by *R*^2^ values, β coefficients and *p*-values, the strongest association was observed in IgG_1_ (β = 2.793, *p* = 1.7E−8), whereas the weakest in IgG_2_ (β = 1.267, *p* = 0.009). With an *R*^2^ of 0.074, a β coefficient of 1.856 and a *p*-value of 9.6E−4, IgG_3_ displayed a slightly stronger association between its agalactosylation and CA125 than that observed in IgG_2_ but weaker than that in IgG_1_.

## Discussion

In the present study, we investigated the EOC-related glycosylation changes in the three most abundant IgG subclasses, namely, IgG_1_, IgG_2_ and IgG_3_. IgG glycosylation in OC was already examined by several research groups including ours (9, 10, 12, 13, 22), demonstrating quantitative changes in galactosylation/agalactosylation and its utility to improve the detection of OC. Nevertheless, previous studies monitored the glycosylation profile of total IgG at the level of enzymatically released N-glycans. Owing to the fact that about 15–20% of IgG molecules might contain additional N-glycosylation sites within variable domains of Fab fragments, this approach generates information that is neither Fc-/Fab-specific nor subclass-specific. In the present study, analysis of EOC-related changes in IgG glycosylation was performed at the level of tryptic glycopeptides, incorporating an affinity separation of IgG_3_ to ensure the subclass specificity of obtained glycosylation profiles and to allow a separate examination of three of the most abundant IgG subclasses.

In accordance with previous OC reports (10, 12, 13, 22), EOC was found to be associated with increased IgG agalactosylation/decreased IgG galactosylation (in this study categorized as monogalactosylation and digalactosylation) in all investigated subclasses. Significantly, these alterations were shown to have relevant biological consequences. Agalactosylated IgG glycoforms are known to act in a pro-inflammatory manner, as they are able to activate the complement system via an alternative pathway (33, 34) and the lectin pathway, after binding to mannose-binding lectin (19, 35). Although molecular regulations of the above-described changes are not yet fully understood, in rheumatoid arthritis, a similar trend of reduced IgG galactosylation was shown to result from decreased activity of galactosyltransferase in IgG-producing plasma B cells (36).

Aside from an EOC-related increase in agalactosylation/decrease in galactosylation observed consistently in all investigated IgG subclasses, we detected some distinct subclass-specific trends. Specifically, glycosylation of IgG_1_ was found to be the most strongly affected in EOC, and its agalactosylation showed the strongest association with the CA125 diagnostic marker. Our observations are therefore in line with the results of Ruhaak et al. (22), who showed that glycopeptides of this most abundant IgG subclass exhibit the highest diagnostic potential in EOC. Furthermore, our study revealed that IgG_1_ undergoes the greatest EOC-related reduction in monogalactosylated and digalactosylated glycopeptides, which might indicate its strongest transformation toward a pro-inflammatory phenotype during EOC development. Interestingly, Saito et al. (37) showed that in gastric cancer patients, a decreased IgG_1_ concentration was significantly correlated with a poor prognosis. As the serum concentration of other IgG subclasses showed no relation to patient outcome, authors concluded that IgG_1_ must play the most important role in tumor immunity. Remarkably, in our study, IgG_1_ was also found to be the only subclass whose fucosylation was significantly increased in EOC patients. Our observations are in agreement with the results of Plomp et al. (30), showing that increased IgG fucosylation is characteristic for individuals with high inflammation, which occurs frequently in OC patients (38). Since IgG core-fucosylation is known to considerably reduce binding to FcγRIIIa and FcγRIIIb receptors, its increase might lead to reduced ADCC activity. As much as this effect may be detrimental to patients, it could be advantageous to cancer by promoting its progression.

Interestingly, the glycosylation profile of IgG_2_, marked by particularly low galactosylation, was found to be distinctly different from that of IgG_1_ and IgG_3_. This observation implies that a common approach of simultaneous analysis of IgG_2_ and IgG_3_ might lead to misleading results. IgG_2_ was previously reported to exhibit the lowest affinity for FcγRs and the lowest ADCC activity among all IgG subclasses (23, 39). Also, as reported by Plomp et al. (30), IgG_2_ glycosylation showed weaker association with markers of inflammation and metabolic health than IgG_1_ and IgG_4_, indicating its rather limited role in inflammation. Additionally, decreased IgG_2_ concentrations were reported in various cancer types, including ovarian, gastric and liver cancer (22, 40, 41), which might indicate that IgG_2_ plays a secondary role in cancer development. In line with all those reports, in our study, IgG_2_ glycosylation traits showed the weakest association with EOC and the CA125 diagnostic marker.

An essential part of our study was devoted to the investigation of IgG_3_-specific glycosylation changes in EOC patients, as to the best of our knowledge, it has not been studied so far. Despite being relatively underrepresented (approximately 5–8% of total IgG), IgG_3_ deserves special attention, as in many ways, it is a unique IgG subclass. Its separation can be accomplished due to a particular feature of its amino acid sequence, that is, the presence of arginine instead of histidine at position 435 that prevents its binding to Protein A (42). Moreover, due to its superior affinity toward activating FcγRs and C1q, IgG_3_ was shown to be the strongest inducer of Fc-mediated effector functions, including ADCC and CDC (23). Despite its discrete character, glycosylation of IgG_3_ is rarely determined individually. Instead, it is investigated in combination with IgG_2_, which, as shown in our study, has a markedly different glycosylation profile. Indeed, in terms of glycosylation and EOC-related glycosylation changes, IgG_3_ was found to more closely resemble IgG_1_ than IgG_2_.

Interestingly, even though an observed EOC-related decrease in IgG_3_ sialylation was statistically insignificant after multiple testing corrections, we could confirm the overall high IgG_3_ sialylation when compared to IgG_1_ and IgG_2_, already reported in the literature (41, 43). Although the molecular regulation of increased IgG_3_ sialylation is not completely understood, it could possibly be related to higher accessibility of IgG_3_ Fc-attached N-glycans for sialyltransferases due to structural differences within its Fc-part (42). Additionally, it was proposed that elevated IgG_3_ sialylation could result from different processing by B-cell-independent sialidases and sialyltransferases present in serum (43, 44). Having in mind that addition of sialic acid acts as a functional switch turning IgG molecule from pro-inflammatory state to an anti-inflammatory state, in physiological conditions (healthy controls), high IgG_3_ sialylation could play a protective role by limiting its excessive inflammatory responses. In turn, a decrease of IgG_3_ sialylation in EOC patients might indicate a disease-related transformation toward a pro-inflammatory phenotype, a phenomenon that was observed for other cancer types (40, 45).

IgG glycosylation has already been investigated in various cancer types, often revealing associations with patient survival rate, response to therapy and diagnostic markers. For instance, in gastric cancer patients, a high level of digalactosylated IgG_2_ glycoforms was shown to be an indicator of better survival (40), whereas an IgG glycosylation-based model proposed by Qin et al. (46) could accurately predict response to neoadjuvant therapy. In turn, in prostate cancer, the ratio of IgG agalactosylated structures to the sum of monogalactosylated and digalactosylated ones strongly correlated with levels of prostate-specific antigen (PSA), which is the most prevalently used prostate cancer biomarker (47). In our study, IgG agalactosylation showed a strong positive association with CA125, the serum routine biomarker. While comparisons between IgG glycosylation and CA125 have already been reported (13, 22), this is the first study showing associations with CA125 in a subclass-specific manner. Interestingly, as agalactosylation of all subclasses was found to significantly associate with the CA125 marker, the strongest relation was observed for IgG_1_ and IgG_3_.

In line with previous reports (27, 28, 48), glycosylation profiles of all three IgG subclasses were observed to alter with increasing age. Interestingly, when compared to other subclasses, glycosylation of IgG_1_, which displayed the most dramatic EOC-related alterations and the strongest association with CA125, was observed to be the least affected by age. This could indicate that age-related glycosylation changes might diminish differences between older healthy controls and EOC patients, thereby hindering disease discrimination.

In conclusion, by analyzing EOC-related glycosylation changes in the three most abundant IgG subclasses separately, our study broadens the current understanding of molecular backgrounds of EOC pathogenesis. Interestingly, while glycosylation alterations of all IgG subclasses were observed to follow similar patterns, EOC-related changes were most pronounced in IgG_1_, which might indicate its particularly important role in OC. As subtle subclass-specific characteristics were also detected, our study highlights the importance of independent analysis of IgG subclasses, particularly in respect to IgG_2_ and IgG_3_ subclasses.

## Data Availability Statement

The datasets generated for this study are available on request to the corresponding author.

## Ethics Statement

The studies involving human participants were reviewed and approved by the Charité – Universitätsmedizin Berlin EA4/073/06 and Medical Association of Hamburg #OB/V/03. The patients/participants provided their written informed consent to participate in this study.

## Author Contributions

VB, EB, and JS contributed to the conception and the design of the study. EB and LO-F coordinated the collection of samples and the database. MW performed the experiments and data analysis. All authors contributed to the manuscript writing and revision, read and approved the submitted version.

## Conflict of Interest

The authors declare that the research was conducted in the absence of any commercial or financial relationships that could be construed as a potential conflict of interest.

## Supplementary Material

FIGURE S1 **(A)** Schematic representation of two-step IgG affinity isolation from serum using Protein A and Protein G Sepharose. **(B)** Coomassie stained SDS-PAGE gel presenting Protein A Sepharose-bound fraction containing IgG_1_, IgG_2_ and IgG_4_, and Protein G Sepharose-bound fraction containing IgG_3_. The band of IgG_3_ heavy chain appears higher in the gel due to its higher molecular mass. **(C)** Western blot analysis of (1) Protein A and (2) Protein G Sepharose-bound fractions using anti-human IgG_3_ antibody, showing negligible IgG_3_ contamination of Protein A-bound fraction.

FIGURE S2 Inter-day reproducibility of the IgG subclass-specific glycosylation analysis. The entire analytical procedure including two-step IgG purification, tryptic digestion, cotton-HILIC enrichment and MALDI-TOF-MS measurement were repeated using the same serum sample on three consecutive days. Relative intensities are shown for the six most abundant glycopeptide structures of IgG_1_, IgG_2_ and IgG_3_. Average is shown as mean ± SD. Mean CV of six presented glycopeptides were 1.87 (IgG_1_), 2.97 (IgG_2_) and 3.11 (IgG_3_), indicating good reproducibility of the method.

TABLE S1 Mean relative intensities and SD values of IgG glycosylation traits in healthy controls and EOC patients. Representations of glycosylation traits are given in terms of Agal (agalactosylation), Monogal (monogalactosylation), Digal (digalactosylation), Sial (sialylation), Bisec (bisecting GlcNAc), Fuc (core-fucosylation).
