# Supplementary figures and images for: Immunoglobulin G Subclass-Specific Glycosylation Changes in Primary Epithelial Ovarian Cancer
Source: Front Immunol. 2020 May 15;11:654. doi: 10.3389/fimmu.2020.00654 (PMC7242562; doi:10.3389/fimmu.2020.00654)

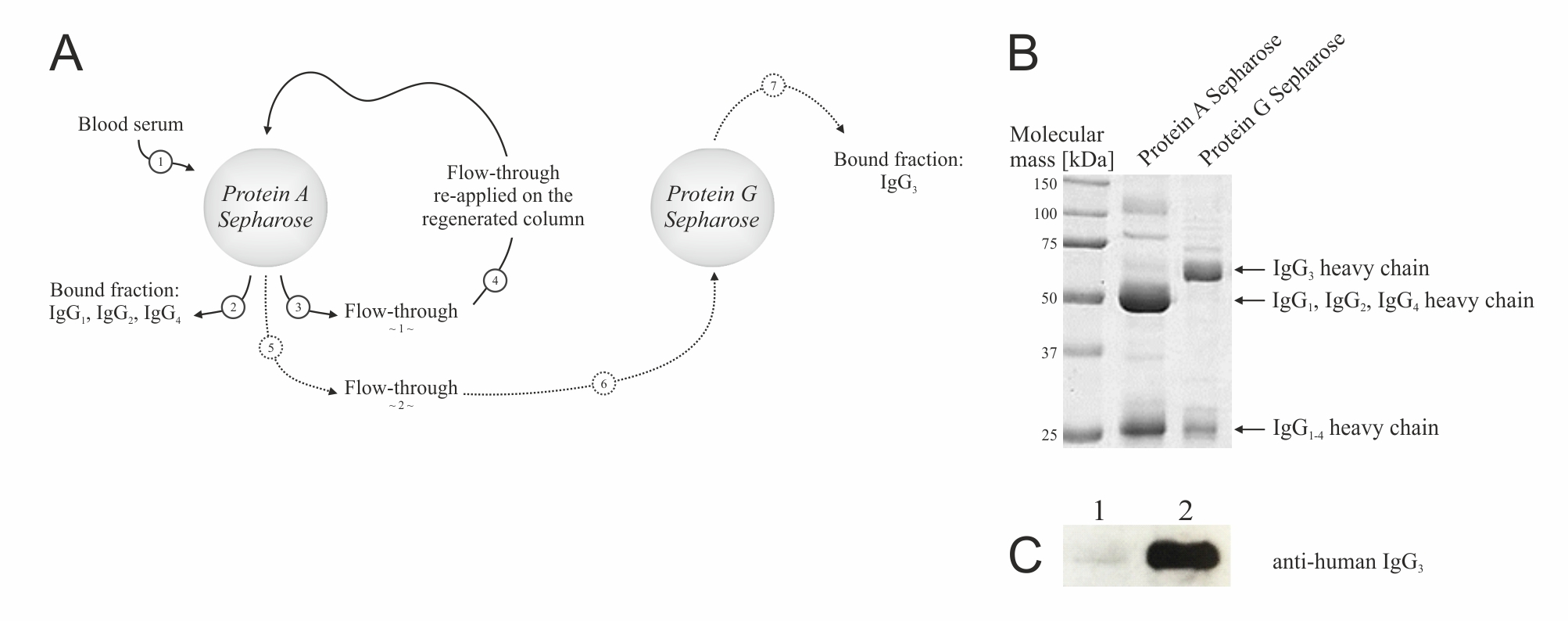

Supplement: FIGURE S1 — (A) Schematic representation of two-step IgG affinity isolation from serum using Protein A and Protein G Sepharose. (B) Coomassie stained SDS-PAGE gel presenting Protein A Sepharose-bound fraction containing IgG1, IgG2 and IgG4, and Protein G Sepharose-bound fraction containing IgG3. The band of IgG3 heavy chain appears higher in the gel due to its higher molecular mass. (C) Western blot analysis of (1) Protein A and (2) Protein G Sepharose-bound fractions using anti-human IgG3 antibody, showing negligible IgG3 contamination of Protein A-bound fraction. [file Image_1.jpg]

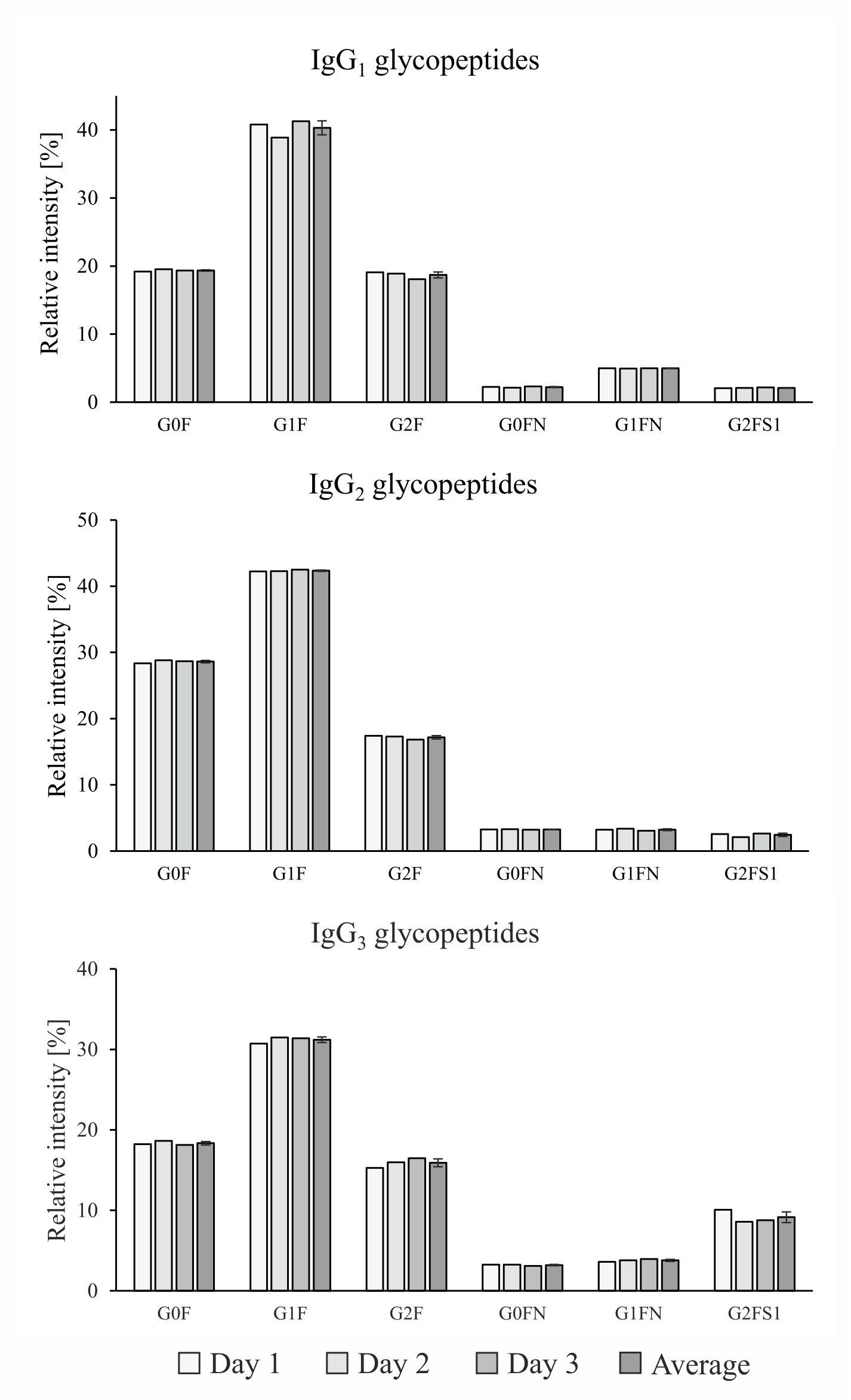

Supplement: FIGURE S2 — Inter-day reproducibility of the IgG subclass-specific glycosylation analysis. The entire analytical procedure including two-step IgG purification, tryptic digestion, cotton-HILIC enrichment and MALDI-TOF-MS measurement were repeated using the same serum sample on three consecutive days. Relative intensities are shown for the six most abundant glycopeptide structures of IgG1, IgG2 and IgG3. Average is shown as mean ± SD. Mean CV of six presented glycopeptides were 1.87 (IgG1), 2.97 (IgG2) and 3.11 (IgG3), indicating good reproducibility of the method. [file Image_2.JPEG]
