# Supplementary material for: Immunoglobulin G Subclass-Specific Glycosylation Changes in Primary Epithelial Ovarian Cancer
Source: Front Immunol. 2020 May 15;11:654. doi: 10.3389/fimmu.2020.00654 (PMC7242562; doi:10.3389/fimmu.2020.00654)
Supplement: TABLE S1 — Mean relative intensities and SD values of IgG glycosylation traits in healthy controls and EOC patients. Representations of glycosylation traits are given in terms of Agal (agalactosylation), Monogal (monogalactosylation), Digal (digalactosylation), Sial (sialylation), Bisec (bisecting GlcNAc), Fuc (core-fucosylation). [file Table_1.DOCX]

| IgG structure | IgG subclass | Healthy  [Mean ± SD] | EOC  [Mean ± SD] |
| --- | --- | --- | --- |
| Agal | IgG_1_ | 31.6 ± 9.6 | 42.7 ± 13.5 |
|  | IgG_2_ | 46.8 ± 11.1 | 52.3 ± 12.7 |
|  | IgG_3_ | 36.4 ± 11.0 | 44.4 ± 15.1 |
| Monogal | IgG_1_ | 49.4 ± 4.2 | 43.8 ± 7.7 |
|  | IgG_2_ | 41.8 ± 5.9 | 38.3 ± 8.1 |
|  | IgG_3_ | 40.4 ± 4.3 | 37.1 ± 6.9 |
| Digal | IgG_1_ | 19.0 ± 7.1 | 13.5 ± 6.4 |
|  | IgG_2_ | 11.9 ± 5.9 | 9.5 ± 5.0 |
|  | IgG_3_ | 23.2 ± 9.2 | 18.4 ± 9.2 |
| Sial | IgG_1_ | 3.2 ± 1.2 | 2.7 ± 1.3 |
|  | IgG_2_ | 5.1 ± 1.7 | 5.1 ± 2.3 |
|  | IgG_3_ | 14.8 ± 6.4 | 12.9 ± 6.3 |
| Bisec | IgG_1_ | 15.5 ± 4.5 | 14.6 ± 3.9 |
|  | IgG_2_ | 11.1 ± 3.5 | 10.3 ± 2.9 |
|  | IgG_3_ | 12.3 ± 3.2 | 11.6 ± 2.9 |
| Fuc | IgG_1_ | 94.5 ± 2.2 | 95.5 ± 1.8 |
|  | IgG_2_ | - | - |
|  | IgG_3_ | 91.4 ± 4.6 | 91.7 ± 4.8 |

**Table S1.** Mean relative intensities and SD values of IgG glycosylation traits in healthy controls and EOC patients. Representations of glycosylation traits are given in terms of Agal (agalactosylation), Monogal (monogalactosylation), Digal (digalactosylation), Sial (sialylation), Bisec (bisecting GlcNAc), Fuc (core-fucosylation).
